# Novel Bi-Factorial Strategy against *Candida albicans* Viability Using Carnosic Acid and Propolis: Synergistic Antifungal Action

**DOI:** 10.3390/microorganisms8050749

**Published:** 2020-05-16

**Authors:** Alejandra Argüelles, Ruth Sánchez-Fresneda, José P. Guirao-Abad, Cristóbal Belda, José Antonio Lozano, Francisco Solano, Juan-Carlos Argüelles

**Affiliations:** 1 Vitalgaia España, S.L. 30005 Murcia, Spain; a.arguellesprieto@um.es (A.A.); ruth.sanchez1@um.es (R.S.-F.); josepedro.guirao@um.es (J.P.G.-A.); 2 Área de Microbiología, Facultad de Biología, Universidad de Murcia, E-30071 Murcia, Spain; 3 Hospital Universitario Sanchinarro, 28050 Madrid, Spain; cbelda@cmcbiotech.xyz; 4 Departamento de Bioquímica y Biología Molecular B e Inmunología, Facultad de Medicina, Universidad de Murcia, E-30100 Murcia, Spain; jalozate@um.es (J.A.L.); psolano@um.es (F.S.)

**Keywords:** carnosic acid, propolis, antifungal action, synergy, *Candida albicans*, biofilms

## Abstract

The potential fungicidal action of the natural extracts, carnosic acid (obtained from rosemary) and propolis (from honeybees’ panels) against the highly prevalent yeast *Candida albicans*, used herein as an archetype of pathogenic fungi, was tested. The separate addition of carnosic acid and propolis on exponential cultures of the standard SC5314 *C. albicans* strain caused a moderate degree of cell death at relatively high concentrations. However, the combination of both extracts, especially in a 1:4 ratio, induced a potent synergistic pattern, leading to a drastic reduction in cell survival even at much lower concentrations. The result of a mathematical analysis by isobologram was consistent with synergistic action of the combined extracts rather than a merely additive effect. In turn, the capacity of SC5314 cells to form in vitro biofilms was also impaired by the simultaneous presence of both agents, supporting the potential application of carnosic acid and propolis mixtures in the prevention and treatment of clinical infections as an alternative to antibiotics and other antifungal agents endowed with reduced toxic side effects.

## 1. Introduction

Natural compounds obtained from plants and other sources have been successfully used for healthcare, cosmetics or food since ancient times, and they represent an important biotechnological target in today’s world. Rosemary (*Rosmarinus officinalis*) is one of the most widely studied plants for this goal, because it contains several terpenoids and other bioactive compounds, of which carnosic acid (CA) is one of the most prominent since it is endowed with both antioxidant and antimicrobial activities [1,2]. Due to these properties, CA has been used successfully in several biotechnological applications and as a food additive to improve the stability of meat [3].

In turn, propolis (PP) is a generic name for a resinous and assorted material produced by honeybees (*Apis mellifera*) from a large number of substances collected from different parts of plants, including buds and exudates, with broad clinical applications [4]. The composition of PP depends on the geographic area and the flora, climate and region covered by honeybees [5,6]. Indeed, more than 300 constituents have been identified and new ones are still being recognized. Therefore, the chemical composition of propolis is not only complex but also highly variable with serious standardization problems [4]. However, the most common constituents of raw PP are typically 50% plant resins, 30% waxes, 10% essential oils, 5% pollen and 5% of other components of a diverse origin and nature. Among the large list of beneficial applications of propolis, the most well-described include anti-inflammatory, antioxidant, immunostimulant, cytostatic, antimicrobial, antitumor and hepatoprotective activities [7,8].

The present work focuses on the application of these two natural extracts, CA and PP, in the development of new antifungal chemotherapies. This field has traditionally received less attention and research effort than an investigation into their bacterial action because (i) a low number of fungal pathogens were thought to exist, most of them of an opportunistic nature; (ii) mycosis were usually superficial infections with good prognosis, except in the case of complications. Nevertheless, this scenario has changed in recent years, during which a dramatic increase in morbidity and mortality caused by septicemic mycosis has been recorded. This septicemia particularly affects patients subjected to invasive surgery, medical implants, or lengthy hospitalization, as well as the immunocompromised chronic population suffering AIDS and other viral diseases [9,10,11].

Moreover, there is growing concern over several fungal species that were classically considered as innocuous, and which are responsible for infectious outbreaks in hospitals [10,12]. In fact, the opportunistic ascomycetous yeast *C. albicans* now represents the fourth most common cause of nosocomial diseases worldwide [11]. Therefore, the search for new, safer, and more potent antifungal compounds is an urgent clinical need [13]. Apart from using known natural or chemically synthesized antifungal agents, which frequently cause unwanted side effects after prolonged treatment, a complementary strategy focuses on the search for novel natural products with effective fungicidal activity. Here, we report on an unexpected potent antifungal effect shown by mixtures of CA and PP. Both are natural agents with certain antioxidant properties, and they are commonly employed and commercialized as food additives or as prodrugs because of their beneficial effects on the human metabolism, without undesirable side effects.

## 2. Materials and Methods

### 2.1. Yeast Strains and Culture Conditions

The *C. albicans* SC5314 standard strain was used throughout this study. This same strain has been employed in recent studies on susceptibility to the antifungal compounds polyenes and echinocandins [14]. Yeast cell cultures (blastoconidia) of this opportunistic pathogen were grown at 37 °C by shaking in YPD medium consisting of 2% peptone, 1% yeast extract and 2% glucose.

### 2.2. Natural Extracts

Carnosic acid is a natural benzenediol abietane diterpene found in rosemary (*Rosmarinus officinalis*) and common sage (*Salvia officinalis*). Dried leaves of rosemary contain around 2% CA. The CA used for this work was provided by Nutrafur S.A. (Murcia, Spain). This preparation was obtained according to previously described methods [3,15,16] with slight modifications to get the highest purification. Purity was assayed by HPLC–UV-DAD using conditions described below. According to that, the content of diterpenes is enriched to reach around 80%, with CA being the most abundant one (around 72%, see Table 1 for composition in other components). This preparation was referred to as CA throughout the current manuscript and dissolved in 98% ethanol until the required concentration for the antifungal assays. However, the possibility of some other components (minor diterpenes or others) could be involved in the antifungal action cannot be totally ruled out.

Concerning propolis, several extracts from different geographic regions were initially tested. For the biotechnological and therapeutic purposes of this study, the best antimicrobial outcomes and availability corresponded to a propolis of Chinese origin, which could be provided in great amounts by Monteloeder local supplier (Parque Empresarial, Alicante, Spain). The raw PP stuffs were ground to a fine powder in a mortar and then dissolved by gentle shaking in prewarmed ethanol (98%, at 55 °C) at the required concentrations for antifungal assays. A control with 98% ethanol was run without any significant antimicrobial effect. Other samples were dissolved in dimethyl sulfoxide for chromatographic analysis (see below). According to these determinations, the total polyphenols content was estimated in the 70%–90% range. Particular details about the complex composition are out of the scope of this study. The main goal of this focused on the use of an antifungal mixture of raw PP and carnosic acid rather than the fractionation and characterization of the PP active compounds. Further studies are underway in order for fractionation and identification of the compounds or fractions responsible for the antifungal action, as well as the effect on other yeast strains.

### 2.3. HPLC

Conditions for analytical HPLC were as described by Benavente-García et al. [17] with slight modifications. Samples of CA or PP (5 mg/mL) were dissolved in DMSO, filtered through a nylon membrane 0.45 mm pore size. 20 µL of solution were injected in a LiChrospher 100-C18 reverse-phase column (250 × 4.0 mm inner diameter) thermostatized at 30 °C. Mobile phase consisted of a gradient from acetonitrile/2.5% acetic acid aqueous solution starting with 5%/95% and finishing with 95%/5% acetonitrile/acidic water. The flux was 1 mL/min. Detection was followed by a Diodo-Array Agilent UV-Vis detector. Usually, chromatographic profiles were monitored at 280, 340 and 370 nm for differentiated identification of phenols and flavonoids.

### 2.4. Determination of Cell Viability

*C. albicans* cultures were grown at 37 °C in YPD until they reached exponential phase (OD_600nm_ = 0.8–1.0) and were then divided into several identical aliquots, which were treated with the concentrations of CA and PP indicated in the Results section. In all series of experiments, control samples with neither extract added were incubated simultaneously. Cell viability was determined in samples diluted appropriately with sterile water by plating in triplicate on solid YPD after incubation for 1–2 days at 37 °C. Between 30 and 300 colonies were counted per plate. Survival percentages were normalized to control samples (100% viability). Colony growth in solid medium was tested by spotting 5 μL from the respective 10-fold dilutions onto YPD agar. Then, the plates were incubated at 30 °C and scored after 24 or 48 h. The polyene Amphotericin B has been included as a positive control of antifungal activity.

### 2.5. Morphological Analysis

After exposure to the different antifungals, cell morphology was recorded with a Leica DMRB microscope using the Nomarsky interference contrast technique. The microscope was equipped with a Leica DC500 camera connected to a PC containing the Leica Application Suite V 2.5.0 R1 software.

### 2.6. Biofilm Formation

In vitro biofilm formation was analyzed on the surface of polystyrene 96-well microtiter plates using previously described methods (Pierce et al. 2014). Briefly, 100 µL of the analyzed *C. albicans* suspension (1.0 × 10^6^ blastoconidia/mL) in RPMI 1640 was allowed to adhere and form a biofilm at 37 °C for 24 h. Following biofilm formation, the medium was aspirated, and non-adherent cells were removed by washing three times with sterile phosphate saline buffer (PBS). Then, CA and PP were added immediately as separate extracts or as mixtures and the biofilms were further incubated for 24 h. The viability of yeast cells within the biofilms was quantified by means of 2,3-bis-(2-methoxy-4-nitro-5-sulfophenyl)-2H-tetrazolium-5-carboxalinide reduction assay (XTT) reduction assay. XTT (Sigma Chemicals) was prepared as a saturated solution at 0.5 g L^−1^ in PBS, filter-sterilized through a 0.22 µm pore size filter, dispensed and stored at –70 °C. An aliquot of the stock solution of XTT (100 µL) was thawed prior to each assay and 10 mM menadione (Sigma Chemicals) in ethanol was added to obtain a final concentration of 25 µM. A 100 µL aliquot of the XTT–menadione solution was added to each well and the plates were incubated for 2 h al 37 °C. The metabolic activity of sessile *C. albicans* cells was assessed quantitatively by measuring the absorbance in a microtiter plate reader (Asys Jupiter) at 490 nm. The tetrazolium salt that accumulated following the reduction of XTT by fungal dehydrogenases was proportional to the number of viable cells present in the biofilm. A base line at 490 nm was run for background subtraction before the measurement of each microplate. Data were expressed as the percentage of metabolic activity in the treated biofilm samples with respect to the 100% (untreated controls).

## 3. Results and Discussion

### 3.1. Single Antifungal Action of CA and PP on C. albicans

**Figure 1:**
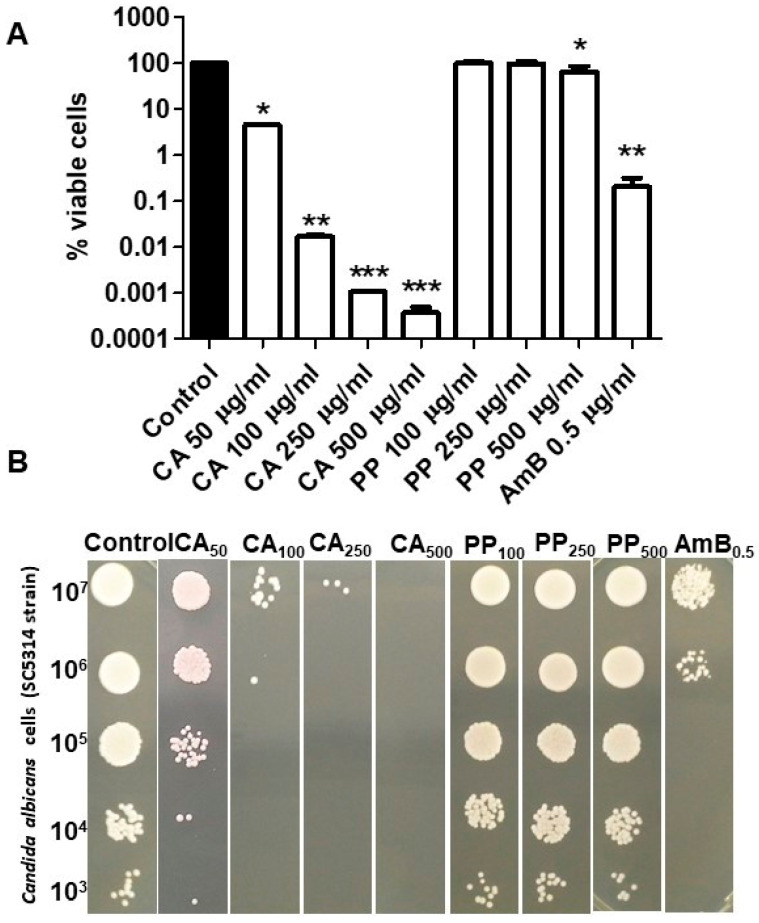
Fungicidal effect caused by the addition of CA or propolis (PP) on the standard strain SC5314 of *C. albicans*. Exponential cells were aliquoted and treated for 1 h at 37 °C with the indicated concentrations of CA and PP. Samples for each treatment and a control (black bar) were spread on YPD plates and the viability was determined by CFU counting (**A**). The macroscopic colonial growth in the different conditions was also recorded in solid media. 10^7^ cells/mL were diluted in YPD and 10-fold dilutions thereof, were spotted in 5 μL onto YPD agar. The plates were further incubated at 37 °C and scored after 24 h (**B**). Amphotericin B (AmB) was included as a positive antifungal control. The data shown are representative of three independent experiments. Statistically significant differences (* = *P* < 0.05; ** = *P* < 0.01; ***, *P* = 0.001) with respect to an untreated control according to Mann–Whitney U test.

Several reports have suggested the existence of noticeable antimicrobial activity in CA and PP extracts against a set of pathogenic bacteria and fungi [3,18,19,20]. Accordingly, we carried out a comparative analysis of this potential antifungal effect against the standard SC5314 strain of *C. albicans* using a range of concentrations similar to that previously chosen in other studies on antimicrobial tests with the same compounds [21,22,23]. In this regard, the concentrations of CA applied were lower than those used for PP. As can be seen in Figure 1A, the fungicidal action of CA in liquid YPD medium was substantial, a large fall in cell viability being observed on exponential *C. albicans* cells after 1 h of exposure to the diterpene. This action was proportional to the applied dose and clearly noticeable even at the lower concentration tested (50 μg/mL), and very pronounced at the highest concentration (500 μg/mL). In turn, the effect of PP was rather weak, and the extract only induced a small but statistically significant degree of cell death after addition of 500 μg/mL (Figure 1A). The parallel assays to measure colony growth on solid YPD plates showed a good correlation with the data obtained in liquid medium (Figure 1B).

The MIC_50_ values for the *C. albicans* SC5314 standard strain in the presence of CA and PP were calculated following the European Committee on Antimicrobial Susceptibility Testing (EUCAST) protocol established for this parameter [24]. The obtained values were 125 μg/mL for CA and 250 μg/mL for PP. These concentrations are within the range previously reported for other *C. albicans* genetic backgrounds of clinical or laboratory origin (EUCAST) [24].

### 3.2. Antifungal Activity of CA and PP Mixtures on C. albicans

**Figure 2:**
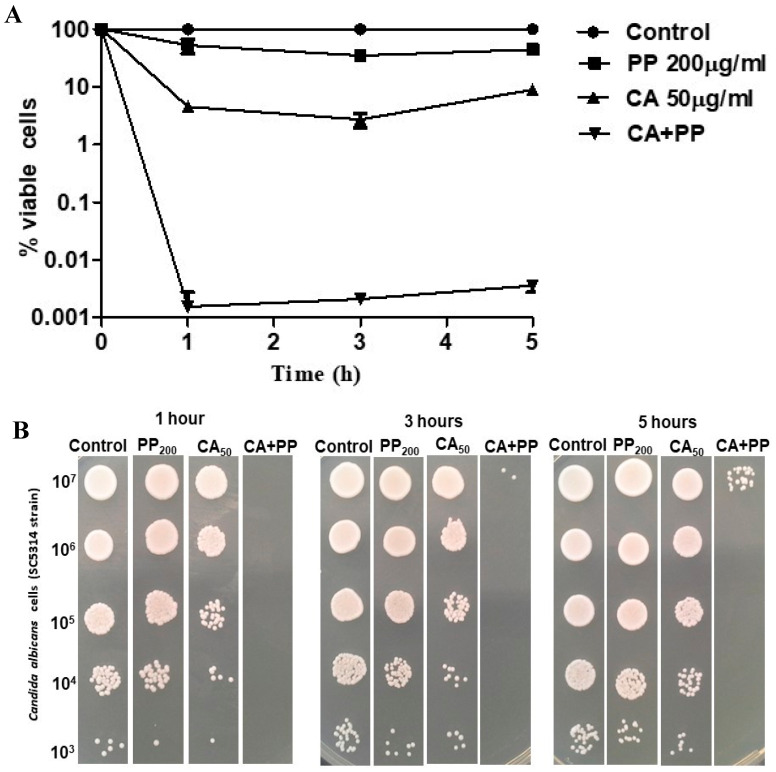
Time-course evolution of cell viability (%) after the addition of 50 μg/mL CA and 200 μg/mL PP, either separately or combined, to cultures of SC5314 strain growing on YPD. After incubation for 1 h at 37 °C, identical samples were harvested and washed at the indicated periods and the percentage of surviving cells in liquid medium (**A**) or the formation of colonies on solid plates (**B**) was determined. The experiments were repeated three times with consistent results. For other details, see Figure 1.

We also performed a more in-depth study regarding the action of both components either as an isolated application or in combination. Different CA:PP proportions were assayed in preliminary experiments (results not shown). Figure 2 shows the degree of *C. albicans* cell death as a function of time (1–5 h) in liquid medium (Figure 2A), as well as a parallel assay of colony growth on solid YPD plates (Figure 2B). Taking into account that CA always displayed higher fungicidal action than PP at identical doses, we chose the lowest concentration of CA (50 μg/mL) and 200 μg/mL of PP to maintain a 1:4 ratio.

The experimental results presented in Figure 2A confirm that CA had greater candidacidal activity than PP, measured in terms of viable cells (CFUs, Figure 2A), the difference being clearly perceptible after only 1 h of treatment, albeit no further reduction was observed at longer times. However, the exposure of *C. albicans* cultures to a combination of CA and PP (1:4) led to a much larger loss (up to five log units) of cell viability compared with the effect of the agents added individually. This pattern was maintained throughout the experiment (Figure 2A), although a modest recovery of cells was evident after 5 h of treatment. The data from the liquid medium showed a very good correlation with the macroscopic growth recorded on solid plates (Figure 2B). Overall, we provide consistent evidence supporting the fact that the combination of CA plus PP induces a fast and strong fungicidal action, which is undoubtedly more effective than the mere addition of one of the substances alone.

If the incubation time is lengthened from 1 h to 5 h, this loss of cell viability appears to be mitigated and a slight but consistent resumption of active growth can be observed rather than the complete elimination of the cultures (Figure 2A,B). It seems that the small number of cells surviving after 1 h was sufficient to start new cycles of bud division. Although a plausible explanation for this residual fraction of viable blastoconidia may come out from an insufficient dose of extracts applied, this should not be the case, because a five-fold increase in the concentration of CA was not dose-response (Figure 1), whereas the weak antifungal effect of PP at 100 μg/mL and 500 μg/mL was rather similar (Figure 1).

As an alternative, the presence of minority cells that possess intrinsic resistance (persister cells) might be operative [25] or the possible inactivation of at least one extract. Whatever the case, further studies are required in order to throw more light on this pattern. Bearing in mind the therapeutic potential of applying a combination of both agents, it is important to get the best formulation for this mixture and identify the intracellular effects on fungal cells during the time of exposure [2,26].

### 3.3. Morphological Changes Induced by Exposure to CA and PP

**Figure 3:**
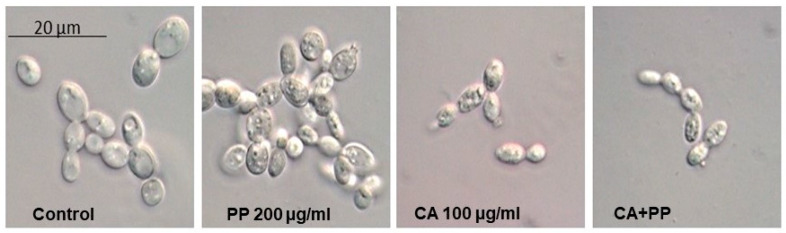
Morphological changes in C. albicans SC5314 blastoconidia induced by the individual addition of CA and PP or in combination. YPD-grown exponential cells (OD_600_ = 0.8) were exposed for 3 h at 37 °C to the indicated concentrations of CA and PP. An untreated sample was maintained as a control. Two similar representative images from each treatment were taken by means of Nomarsky interferential contrast.

The hypothetical morphological changes recorded in growing SC5314 cells upon exposure to both extracts for the intermediate time of 3 h, were monitored by optical microscopy (Figure 3). Similar observations were also visible at longer times (5 h, not shown). For this purpose, identical cell samples were fixed with formalin and kept at 4 °C until their microscopical examination. The Nomarsky interferential contrast confirmed the reduction in the number of viable cells, particularly after the combined action of CA and PP (Figure 3). In this analysis, a concentration of 100 μg/mL was used, since a lower dose (50 μg/mL) did not cause alterations in the external yeast shape, despite it inducing a significant degree of cell killing (Figure 1). A morphological inspection indicated an increase of inner granularity in the samples treated with PP respect to the control. However, and somehow surprisingly, no appreciable cell lysis could be observed, as it occurs in the same strain exposed to 0.5 μg/mL Amphotericin B or 0.1 μg/mL Micafungin [14], indicating different mechanisms of action of the antibiotic and the natural agents herein studied. In turn, the addition of CA, alone or combined with PP, promoted a clear diminution of the cell volume (Figure 3).

### 3.4. Isobologram Plot: Mathematical Demonstration of Synergism

The analysis of the results obtained by single treatments with CA or PP in comparison to combined mixtures reveals a remarkable reduction of the required doses of both compounds to get a similar degree of cell death in cultures of *C. albicans* (Figure 1 and Figure 2). Thus, the collated data from cell survival clearly points out the existence of a reciprocal stimulation of the fungicidal activity due to the combination of CA and PP.

**Figure 4:**
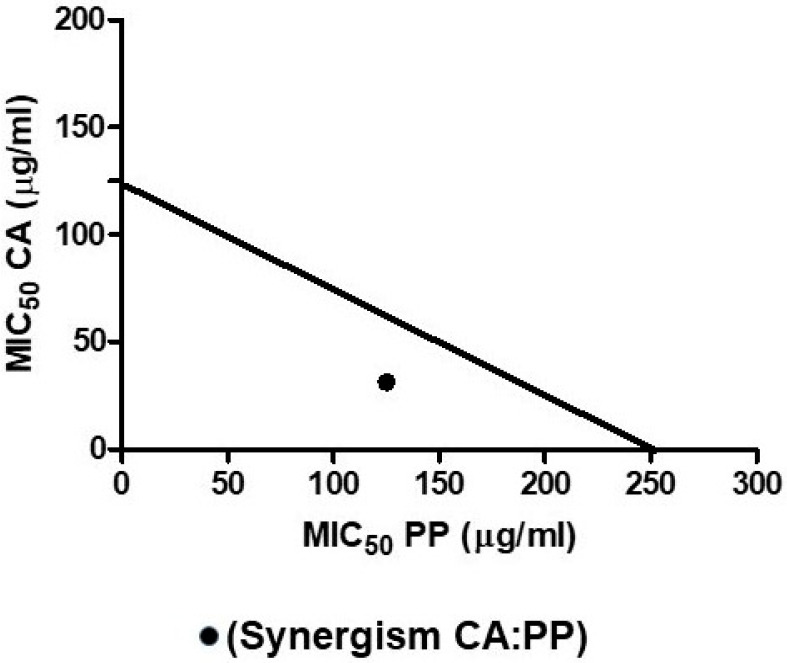
Isobologram plot showing the synergistic action of CA and PP on the *C. albicans* SC5314 strain. The individual MIC_50_ values calculated for CA and PP are represented on the Cartesian axes. The combination of both compounds gives rise to a point that falls inside in the right triangle obtained by drawing the line that joins the MICs of the individual agents.

Nevertheless, this outcome, which could be of importance for the potential therapeutic use of the agents, still needed to be demonstrated by a quantitative mathematical approach. The synergistic cooperation between two compounds endowed with antimicrobial activity has been carefully considered previously [27], utilizing isobolograms to confirm (or not) the strength of the effect. According to this, the MICs for each individual compound should be plotted on the corresponding Cartesian axes for their respective concentrations (Figure 4). The combined MIC value corresponds to the intersection point recorded after plotting. Thus, the action of two compounds would be additive if this point lies on the line that joins the individual MICs. However, the action would be synergistic when that point fits within the right triangle obtained, and the effect would be antagonistic if the point falls outside this triangle. In our case, the plot took as reference the corresponding MIC_50_ values calculated previously for the separate additions of CA and PP represented on the respective Cartesian axes (Figure 4). The point obtained after the supply of a mixture of both compounds corresponds to a concentration of 31.25 μg/mL (CA) and 125 μg/mL (PP) acting on growing SC5314 *C. albicans* cultures (Figure 4). This clearly fits inside the corresponding right triangle, confirming that the combined action of CA plus PP at the stated ratio of 1:4 conveys a strong synergic fungicidal action.

### 3.5. The Capacity to Form Biofilms Is Impaired by the Addition of CA and PP

The infections provoked by *Candida albicans* that give rise to the formation of structured biofilms have important clinical repercussions, particularly among the immunocompromised patients [28,29]. However, the therapeutic options available for the successful treatment of drug-resistant biofilms are scarce and new strategies need to be designed to address this medical problem [28,30]. We have previously examined the efficacy of the antifungals Amphotericin B and Micafungin in connection with the trehalose biosynthetic pathway in *C. albicans* and *C. parapsilosis*, with a moderate degree of therapeutic success [14].

**Figure 5:**
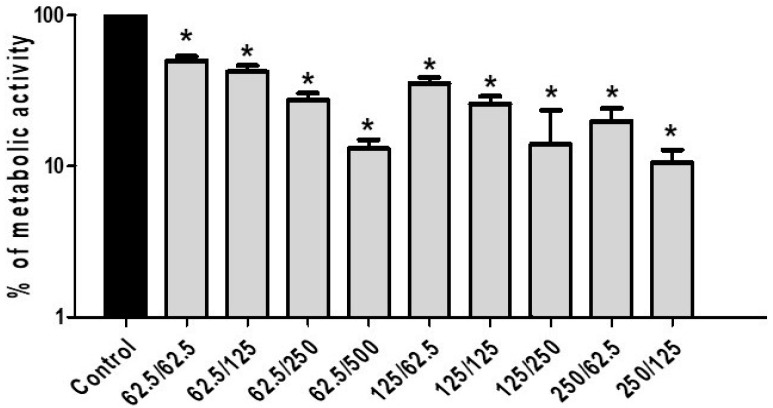
Biofilm formation capability recorded by different combinations of CA and PP in *C. albicans* SC5314 strain. The metabolic activity of the formed biofilms was quantified by the XTT reduction assay (see Methods). The results are expressed as the mean + standard deviation of two experiments with six replications for each group. The concentrations of CA and PP are expressed as µg/mL. Statistically significant differences (* = *P* < 0.05) were recorded with respect to an untreated control according to the Mann–Whitney U test.

The action of CA and PP added individually or in combination, on the percentage of cell viability inside the preformed biofilms was assessed using the XTT reduction assay described in the Methods section. In these experiments, three concentrations of CA were used with the concomitant addition of increasing doses of PP. In these particular series, the CA:PP relation varied from 1:8 to 4:1 due to the particular characteristics of the biofilms. As can be seen in Figure 5, incubation of SC5314 sessile cells for 24 h in the presence of increasing CA:PP ratios induced a notable reduction in the metabolic activity and impaired the degree of biofilm production. Of the different formulations tested, a similar maximum degree of inhibition was achieved with the CA:PP ratios of 62.5:500 and 125:250 (Figure 5). These data demonstrate that the synergistic action of CA and PP has a direct and progressive inhibitory effect on the capacity of *C. albicans* to form biofilms. They also reinforce the potential therapeutic application proposed for the assayed formula.

## 4. Conclusions

Herein, we present preliminary but conclusive evidence consistent with a synergistic fungicidal action induced by the combination of two natural compounds, CA and PP. These agents are described elsewhere as antioxidants [3,18,21] and they have been used with some beneficial effects on animal cells due to their antioxidant activity and the capacity to scavenge ROS. However, the redox balance inside cells is complex, and some well-founded opinion reports are putting in doubt the widely assumed scientific belief that antioxidants agents are always beneficial for living cells [31]. Recently, some reports suggest that under specific conditions, the simultaneous presence of two antioxidants could result in an opposite cytotoxic action due to the induction of a high intracellular oxidative stress impairing mitochondrial function and aerobic metabolism [32,33]. Presumably, the synergistic action herein reported would be related to intracellular impairment of the antioxidant–prooxidant balance caused by both agents. In this regard, quinone reductases are crucial enzymes in the maintenance of the cellular redox balance, especially quinone reductase 2 [34].

Taken together, our results support the continued search for more natural bioactive molecules with the purpose of their application in clinical therapies against *C. albicans* and possibly other harmful pathogenic fungi. This strategy would enable us to avoid the undesirable noxious side effects triggered by some conventional antifungals and could surmount their low selective toxicity due to the eukaryotic nature of fungal cells. Indeed, our previous approaches testing accredited compounds, like validamycin A or resveratrol, yielded quite modest antimicrobial outcomes [35,36]. Further studies are currently underway in order to unravel the mechanisms involved in the synergistic fungicidal activity of CA and PP mixtures.

## 5. Patents

A patent resulted from the work here reported. Title: Synergistic composition comprising propolis and carnosic acid for use in the prevention and treatment of candidiasis. Authors. J.A. Lozano, J.C. Argüelles, A. Argüelles, R. Sánchez-Fresneda and J.P. Guirao-Abad. Reference: Europe (EP 3272344), USA (10,272,120 B2), Canada (2,975,047). Date of concession: 01/31/2019.

## Figures and Tables

**Table 1:** Composition of the semipurified carnosic acid (CA) used in this study.

Component	%
Carnosic acid	70%–75% (mean 72%)
Minor diterpenes (carnosol, rosmanol, epirosmanol and 12-methyl carnosate)	3%–7%
Other plant lipids	5%–10%
Plant carbohydrates	2%–5%
Proteins	0%–0.5%
Water	2%–4%
Mineral salts	1%–2%
Other plant materials	2%–4%
